# First year of ongoing invasive *Haemophilus influenzae* type b outbreak among middle-aged people who use drugs and people who experience homelessness in Hamburg, Germany, September 2024 to August 2025

**DOI:** 10.2807/1560-7917.ES.2026.31.35.2600134

**Published:** 2026-09-03

**Authors:** Silja Bühler, Vanessa Piechotta, Martin Noldt, Christine-Maria Hartog, Caroline Peine, Cornelius Rau, Claudia Siffczyk, Katja Kajikhina, Ole Wichmann, Iris Meier, Maja V Nielsen, Melanie Lang, Amra Arif, Emma Edel, Jennifer Gabbert, Claudia Ruscher, Alexandra von Reiswitz, Jenny Schreiber, Somayyeh Sedaghatjoo, Silver A Wolf, Torsten Semmler, Heike Claus, Viktoria Schönfeld, Thiên-Trí Lâm

**Affiliations:** 1 Institute for Hygiene and Environment, Division for Infectious Disease Epidemiology, Hamburg, Germany; 2 Department for Infectious Disease Epidemiology, Robert Koch Institute, Berlin, Germany; 3 Postgraduate Training for Applied Epidemiology (PAE), Department for Infectious Disease Epidemiology, Robert Koch Institute, Berlin, Germany; 4 ECDC Fellowship Programme, Field Epidemiology path (EPIET), European Centre for Disease Prevention and Control (ECDC), Stockholm, Sweden; 5 Public Health Department Wandsbek, Hamburg, Germany; 6 Public Health Department Hamburg-Mitte, Hamburg, Germany; 7 Public Health Department Altona, Hamburg, Germany; 8 Public Health Department Eimsbüttel, Hamburg, Germany; 9 Public Health Department Harburg, Hamburg, Germany; 10 State Office for Health and Social Affairs Mecklenburg-Vorpommern, Rostock, Germany; 11 State Office for Health and Social Affairs (SOHSA), Berlin, Germany; 12 State Ministry of Health, Social Affairs and Integration, Department of Public Health, Hamburg, Germany; 13 Genome Competence Centre (MF1), Robert Koch Institute, Berlin, Germany; 14 Integrated Genomic Surveillance, Robert Koch Institute, Berlin, Germany; 15 National Reference Centre for Meningococci and Haemophilus influenzae, Institute for Hygiene and Microbiology, University of Würzburg, Würzburg, Germany

**Keywords:** Haemophilus influenzae b, outbreak, drug use, homelessness

## Abstract

We report an ongoing outbreak of invasive *Haemophilus influenzae* type b (Hib) disease among people who use drugs (PWUD) and people experiencing homelessness (PEH) in Hamburg, Germany. Between 1 September 2024 and 31 August 2025, 20 adult Hib cases were reported. The cases were either residents of Hamburg (16/20) or stayed in Hamburg during the exposure period (4/20). Whole genome sequencing identified a link between 18 cases (median age 50 years (range 26–77); six female, 12 male, three died). An epidemiological link (i.e. drug consumption in the open drug scene) could be established for 15 of the 18 cases. Control measures included post-exposure chemoprophylaxis for close contacts (n = 4), vaccination offers (n = 69 doses administered by 31 August 2025) and an information campaign targeting PWUD, PEH and staff members of addiction support centres. We describe the first Hib outbreak among adults in Europe and document drug use and homelessness as suspected risk factors. Based on these findings, national vaccination recommendations issued by the Standing Committee on Vaccination in Germany (STIKO) were updated to include vaccination and post-exposure prophylaxis recommendations in outbreak settings for PWUD and PEH. Control measures are currently being evaluated and expanded.

**Key public health message**

**What did you want to address in this study and why?**

*Haemophilus influenzae* type b (Hib) is a bacterium that can cause severe and life-threatening illness. It typically occurs in children, while adults are considered immune. However, we identified several severely ill adults with Hib disease in Hamburg, northern Germany over a period of 12 months. We were looking for reasons that made adults vulnerable to Hib and aimed to prevent others from acquiring the disease.

**What have we learnt from this study?**

We found that most of the affected 20 adults were male, used drugs (especially inhalants), had existing health problems and around half experienced homelessness. We suspect that besides existing health problems, drug use and factors associated with homelessness weakened their immune system and enhanced vulnerability to Hib. Sharing equipment for inhaling drugs could have further increased vulnerability.

**What are the implications of your findings for public health?**

We suspected inhalant drug use and homelessness to be risk factors for increased vulnerability to Hib. As Hib usually does not affect adults, there was no available public health guidance on how to protect these persons at risk. This outbreak investigation prompted an update of the national vaccination recommendations in Germany, which now recommend Hib vaccination in outbreak settings among specific vulnerable populations.

## Background

*Haemophilus influenzae* (Hi) is a Gram-negative bacterium that colonises the human nasopharynx. Besides respiratory tract infections it may cause invasive infections such as pneumonia, septicaemia or meningitis. The main mode of transmission is via respiratory droplets and secretions. There are six capsular serotypes (a–f) and unencapsulated strains (nontypeable Hi (NTHi)). Today, the latter dominate the epidemiology of invasive Hi infections in Germany.

Detection of Hi from blood and cerebrospinal fluid (CSF) is notifiable to local health authorities (LHAs) according to Paragraph 7 of the German Infection Protection Act (Infektionsschutzgesetz (IfSG)). As most primary diagnostic laboratories do not perform serotyping, they are encouraged to send isolates to the National Reference Centre for Meningococci and *Haemophilus influenzae* (NRZMHi). The NRZMHi receives isolates from around 80–85% of yearly reported cases and subsequently ascertains serotype.

Invasive Hi infections mainly occur in young children (< 5 years) and older adults (≥ 65 years). Vaccines are only available against the Hi type b (Hib). Prior to 1990 when the Hib conjugate vaccine was introduced into the routine German childhood immunisation programme, Hib was a major cause of invasive infections in children aged under 5 years. With Hib vaccination coverage in young children remaining between 75 and 81% over the past 15 years, invasive Hib disease has become rare in Germany [1]. An average of 21 invasive Hib cases were notified in Germany per year from 2001 to 2024 [2].

Between 1 January 2001 and 31 August 2024, 10 Hib cases were reported in Hamburg, corresponding to approximately 0.4 cases per year. Between 1 September 2024 and 31 August 2025, 16 Hib cases were reported, which corresponded to a 32-fold increase.

Globally, outbreaks of invasive Hi disease are rare. Disease transmission caused by NTHi has been mainly described in care facilities for elderly people [3], and, in recent years, immunodeficient persons have been reported to be at higher risk of NTHi [4]. Outbreaks of invasive Hib disease are infrequent in the post-Hib vaccine era and have previously only been reported in children in Europe.

The Standing Committee on Vaccination in Germany (STIKO) is the mandated commission to issue national recommendations on vaccination and post-exposure prophylaxis. The STIKO recommendations for Hib were developed for the protection of unvaccinated children and immunocompromised persons. Before this outbreak, post-exposure chemoprophylaxis was recommended by STIKO for close contacts of symptomatic cases (face-to-face contact or exposure to oropharyngeal secretions) if a household or childcare facility contact included unvaccinated children under 5 years or immunocompromised individuals. Rifampicin was considered the prophylactic agent of choice. However, these STIKO recommendations were not applicable to outbreak settings in other population groups.

## Outbreak detection

On 17 January 2025, the NRZMHi reported the detection of three invasive Hib cases in middle-aged adults in Hamburg with notification dates less than 14 days apart. The LHAs in Hamburg had received notifications between 11 December 2024 and 2 January 2025. The LHAs subsequently informed the State Institute for Hygiene and Environment (IHE), Hamburg. The IHE then informed the State Ministry of Health, Social Affairs and Integration (together, these institutions constitute the Hamburg State Health Authority (SHA)). The Hamburg SHA is one of the 16 SHAs in Germany. In parallel, the NRZMHi reported the incident to the National Public Health Institute, the Robert Koch Institute (RKI). Initial investigations by the LHAs revealed that in addition to the unusual occurrence of invasive Hib infections in adults, all three cases were people who use drugs (PWUD) and people experiencing homelessness (PEH). This initiated further investigations, first in Hamburg and subsequently in other federal states of Germany.

Here, we describe the first year of a community-based outbreak of invasive Hib disease among adults in Hamburg (September 2024 to August 2025) and include case-finding strategies, case descriptions, carriage prevalence testing, genotyping and the implementation of control measures to prevent further cases in the light of scarce guidelines or evidence.

## Methods

### Data sources

Epidemiological information on notified Hib cases is collected through routine surveillance in accordance with the IfSG. Additional information on epidemiological characteristics investigated as potentially associated with the outbreak (e.g. comorbidities, Hib vaccination, housing status and drug use) is actively collected by LHAs, which contact possible cases, their relatives, treating physicians or other known contacts. Cases considered to be associated with the outbreak are defined and classified as described in Table 1.

**Table 1:** *Haemophilus influenzae* type b case definition and classification, Hamburg, Germany, 1 September 2024–31 August 2025

**Case definition**	
Time/exposure period	Notification between 1 September 2024 and 31 August 2025
Place	Residence or temporary stay in Hamburg during the exposure period
Person	Invasive Hib disease in accordance with national reference definition [29]
**Case classification**	
Confirmed case	Infection with the outbreak strain i.e. genomic cluster established by WGS with ≤ 5 allele differences
Probable case	Hib infection without available WGS data
Possible case	Infection with encapsulated Hi without available serotyping or WGS data

Hi: *Haemophilus influenzae*; Hib: *Haemophilus influenzae* type b; WGS: whole genome sequencing.

## Epidemiological investigations

### Case finding

To identify outbreak cases, active case-finding was initiated at local, regional and national level.

All Hib cases and Hi cases without serotype information notified in Hamburg between 1 September 2024 and 31 August 2025 were subsequently reviewed. Responsible LHAs were contacted via SHA to gather information on comorbidities, vaccination status, housing situation, drug use, clinical manifestation and serotype. Information was obtained by e.g. contacting cases, their close friends or relatives, treating physicians, addiction support centres and supervised drug consumption facilities, and by reviewing hospital records. We informed hospitals, the central prison, supervised drug consumption facilities and housing facilities for PEH in Hamburg. In addition, all Hib cases notified in Germany since 1 September 2024 were followed up by the SHAs regarding possible stays in Hamburg during the exposure period, as well as the patient characteristics described above.

All 16 German SHAs were informed and regularly updated on the spike of invasive Hib disease through the weekly epidemiological teleconference of public health services in Germany hosted by the RKI (‘Epilag’ [5], initial report on 28 January 2025). They were asked to support additional investigations by collecting serotype information as well as possible epidemiological links to Hamburg, for any Hi case notification.

The State Health Authorities advised LHAs to contact primary diagnostic laboratories to submit available Hi samples (culture and clinical samples) to the NRZMHi for further analysis (described in the laboratory methods below). This included both previous cases, for whom samples had not yet been forwarded, as well as newly detected cases.

To account for possible under-reporting, the Institute of Legal Medicine in Hamburg was contacted by the SHA to investigate potentially related cases among deceased people who had experienced homelessness and those with unknown causes of death.

## Microbiological and molecular investigations

### Investigation of *Haemophilus influenzae* type b carriage in a long-term housing facility for people experiencing homelessness

Two cases were identified as staying in the same long-term housing facility for PEH, with no known direct contact between cases during the duration of contagiousness. Following this, all residents and staff members of the housing facility were invited to participate in an investigation of pharyngeal carriage of Hi. The investigation aimed to: (i) detect possible Hib carriage in an unclear contact situation, and (ii) develop a working definition of close contacts in housing facilities for PWUD and PEH through exploration.

Staff members of the housing facility were informed by the LHA 5 days before the swab investigation by phone, email and an information leaflet, which was handed out to all residents within the facility by the staff. Furthermore, previous experiences from studies in similar settings [6] have shown that face-to-face communication with residents by social workers from the facility was deemed the most important way to spread the information among the residents. Pharyngeal swabs were taken from volunteering participants by public health officers after verbal consent was obtained on 10 February 2025. Swabs were directly streaked onto selective agar plates and incubated for 2 days at the IHE. After incubation, suspected *Haemophilus* spp. were sent to the NRZMHi for species identification and subsequent serotyping.

### Serotyping and genomic analysis

The NRZMHi supplemented the epidemiological investigations by serotyping all submitted Hi isolates.

Microbiological data on laboratory-confirmed invasive Hi cases are routinely reported by primary diagnostic laboratories to the LHAs. Corresponding Hi isolates are sent on a voluntary basis to the NRZMHi as part of the national laboratory surveillance programme. Submitted isolates are serotyped upon species confirmation by reference methods as previously described [7]. Outbreak strains were defined as a species-confirmed invasive *Haemophilus influenzae* type b isolates that did not differ by more than five core genome multilocus sequence typing (cgMLST) alleles.

The NRZMHi additionally performed molecular typing directly from patient CSF in cases where no isolate was available and diagnosis was made by PCR. In these cases, encapsulation was detected by amplification of the caspule export gene *bexA* (NHS Meningitidis Real-TM, Sacace Biotechnologies, Como, Italy).

*Haemophilus influenzae* type b isolates considered possibly related to the outbreak (see case definition, Table 1) and all Hib isolates from all other federal states since 1 September 2024 were selected for whole genome sequencing (WGS). Genomic DNA was extracted using the Promega Wizard Genomic DNA Purification Kit (Promega GmbH, Walldorf, Germany) at the NRZMHi. Unique dual-indexed libraries were generated using the Nextera XT DNA Library preparation kit (Illumina, San Diego, United States (US)) according to the manufacturer’s instructions, with the exception that reaction volumes were reduced by 50% at the Genome Competence Centre at the RKI. Sequencing was performed on an Illumina NextSeq 2000 platform using a 2 × 150 bp paired-end configuration, targeting 1 million paired-end reads per sample. The resulting DNA sequences were analysed at the NRZMHi by cgMLST using the Bruker MBioSEQ Ridom Typer (Ridom GmbH, Münster, Germany) as previously described [8]. Minimum spanning trees (MSTs) were constructed from cgMLST allelic profiles using MBioSeq Ridom Typer. Pairwise allelic distances were determined based on shared loci only, with loci containing missing allele calls in one or both isolates excluded from the calculations.

## Results

### Epidemiological investigation

**Figure 1:**
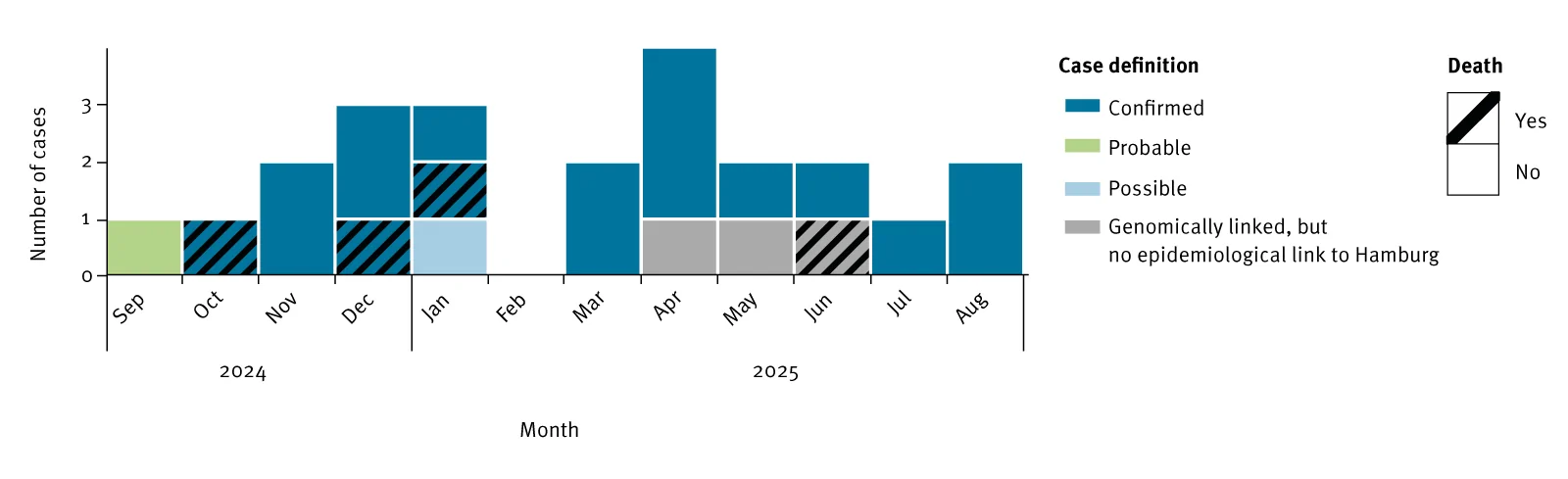
*Haemophilus influenzae* type b cases with residence or temporary stay in Hamburg and further genomically linked cases, Germany, 1 September 2024–31 August 2025

Between 1 September 2024 and 31 August 2025, a total of 46 Hib and 205 Hi cases without serotype information were notified in Germany. Twenty of the Hib cases were considered possibly related to the outbreak because of a known residence or temporary stay in Hamburg during their likely exposure period (Figure 1). Their places of residence were located across four federal states in northern Germany. Eighteen cases were classified as confirmed cases; they had a median age of 50 years (range 26–77) and three died. The most common clinical manifestation was pneumonia (15/18 cases). A high proportion of cases had a history of drug use (15/18), were male (12/18), experienced homelessness (10/18) and had various comorbidities (10/17; information was missing for one case). Information on previous vaccination status was limited and none of the six cases with information on vaccination status had received a Hib vaccination in the past. Further characteristics of confirmed cases are summarised in Table 2.

**Table 2:** Characteristics of confirmed *Haemophilus influenzae* type b cases with residence or temporary stay in Hamburg during the exposure period, Germany, 1 September 2024–31 August 2025

Characteristics		n	%
Total number^a^		18	100
Median age, years (range)		50 (26–77)	
Sex	Female	6	33.3
	Male	12	66.7
Federal state	Berlin	1	5.6
	Hamburg	14	77.8
	Lower Saxony	1	5.6
	Mecklenburg-Western Pomerania	2	11.1
Drug use		15	83.3
Experiencing homelessness		10	55.6
Comorbidities^b^		10/17	58.8
Previous Hib vaccination		0/6	0.0
Clinical manifestation	Pneumonia	15	83.3
	Sepsis	11	61.1
	Meningitis	1	5.5
Outcome	Hospitalisation	18	100.0
	Intensive care unit	10	55.6
	Death	3	16.7

Hib: *Haemophilus influenzae* type b.

^a^ Total number is 18 cases, if not indicated otherwise.

^b^ Information on comorbidities was not systematically collected and reported conditions were not verified. The following comorbidities were reported: HIV (n = 3), hepatitis C (n = 6), chronic obstructive pulmonary disease (COPD) (n = 2), coronary heart disease (n = 2), psychiatric disorder (n = 4), deep vein thrombosis (n = 1), cachexia (n = 1), epilepsy (n = 1), obesity (n = 1), diabetes mellitus (n = 2), asthma (n = 1).

An epidemiological link (drug use in the open drug scene) could be established for all but three confirmed cases. Information on the mode of drug consumption was not systematically collected. For 10 of 15 cases for whom drug use was reported, details of drugs consumed were provided by cases or treating physicians. Use of inhalant drugs, in particular crack / cocaine and methadone substitution (both 8/10) were the most commonly reported substances. Other cases reported the use of heroin, cannabis and other or unspecified opiates.

Eight individuals were known to have contact with the open drug scene near a low-threshold addiction support centre / supervised drug consumption facility close to the city centre and Hamburg Central Station. Several cases reported sharing drug-use equipment (e.g. crack pipes, joints). Further, two confirmed cases resided in the same long-term housing facility for PEH; however, no direct contact between these cases was reported at the facility. Both of these cases had contact with the open drug scene.

No additional cases among people with deaths of unknown cause were identified through investigations at the Institute of Legal Medicine in Hamburg during the above-mentioned period.

## Microbiological investigations

### Investigation of *Haemophilus influenzae* type b carriage in a long-term housing facility for people experiencing homelessness

After confirmation of two Hib cases in a housing facility for PEH, 42 of 111 residents (38%) and two of three staff members consented to a pharyngeal swabbing by officers of the responsible LHA. After incubation, *Haemophilus* spp. were detected in the samples of five participants. Of these, four isolates were identified as NTHi and one as *Haemophilus parainfluenzae.* None of the participants had a positive Hib swab sample.

### Molecular characterisation and whole genome sequencing

**Figure 2:**
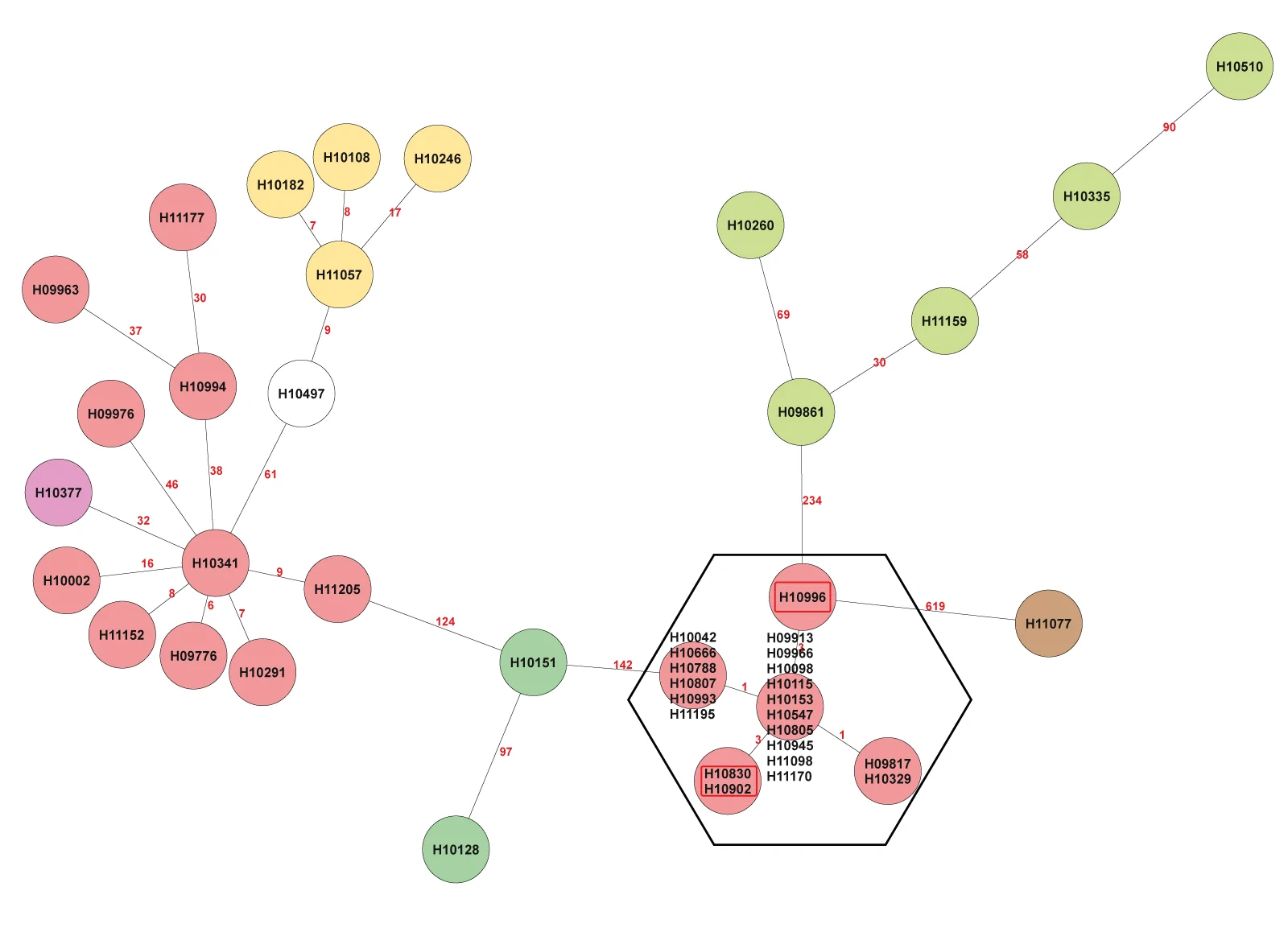
Minimum spanning tree based on whole genome sequencing of all available isolates of notified *Haemophilus influenzae* type b cases in Germany, 1 September 2024–31 August 2025

One CSF sample from a possible outbreak case was submitted to the NRZMHi. Using real-time PCR, encapsulation was detected in this case. *Haemophilus influenzae* isolates from 18 confirmed cases submitted to the NRZMHi were subjected to WGS. All these isolates were assigned to multilocus sequence type (ST) 6 and clustered together in a minimum spanning tree according to cgMLST analysis with zero or one allele differences between the isolates (Figure 2). Phylogenetic investigation showed a clear genetic distinction of the outbreak strain.

Each identification number (ID) represents a *Haemophilus influenzae* type b case. Numbers displayed in red indicate allele differences between isolates. Nodes containing multiple IDs represent genomic sequences with a distance of 0. Node colours correspond to sequence type (ST) (i.e. ST6 shown in red, ST92 in green, ST 95 in light green, ST190 in yellow, ST1721 in brown, ST unknown for purple and white). Nodes in the black hexagon represent outbreak-associated isolates based on genomic criteria. Cases in the red boxes represent isolates within the genomic cluster with no epidemiological link to Hamburg.

Genomic sequences of outbreak strains were compared with Hib strains originating from other federal states in Germany notified during the investigation period (n = 27). Of these, three cases fulfilled the defined genomic criteria (≤ 5 allele differences to the outbreak cluster), but no link to Hamburg or other epidemiological association with the outbreak (drug use or contact with PWUD or PEH) could be ascertained. Two cases were children aged 0 and 1 year (one unvaccinated, one with unknown vaccination status). One was a woman in her late 60s who had a pre-existing condition (malignoma) and died from Hib disease with unknown vaccination status.

Additionally, 10 non-outbreak strains were also allocated to ST6, but demonstrated ≥ 162 allele differences from the outbreak strains and ≥ 6 allele differences from each other. The other non-outbreak strains belonged to ST92 (n = 2), ST95 (n = 5), ST190 (n = 4), ST1721 (n = 1), and unknown ST (n = 2).

## Outbreak control measures

Regular and extraordinary meetings between LHAs, SHAs, RKI and NRZMHi were held on the status of the outbreak investigations and to discuss possible prevention and control measures. National outbreak control protocols were reviewed, in particular STIKO recommendations. As the STIKO recommendations were limited in applicability in the presented outbreak context, additional international guidelines from the United Kingdom Health Security Agency (UKHSA) and British Columbia Centre for Disease Control (BCCDC) were considered to inform decisions on measures to prevent secondary cases and control the outbreak. These international guidelines have been recently updated and could be applied to the outbreak setting. An overview of consulted recommendations is appended in the Supplement. A web-based exchange with staff from BCCDC was established to learn how measures were developed and implemented during a similar Hib outbreak among PWUD and PEH in British Columbia, Canada and to discuss their experiences. Additionally, regular outbreak updates were presented to STIKO to assess whether national recommendations on prevention measures needed to be updated.

European countries were asked about their experiences with Hib outbreaks via Epipulse*,* an online portal hosted by the European Centre for Disease Prevention and Control for European public health authorities and global partners to rapidly share, collect, analyse and discuss infectious disease data. None of the countries had experiences with similar outbreaks at that time.

### Contact tracing and post-exposure chemoprophylaxis

Contact tracing was complicated by a high degree of anonymity and multiple social contacts in the target population, including frequent changes in people’s whereabouts, unknown or non-disclosed personal information of possible contacts and language barriers. In addition to the STIKO recommendations, close contacts were defined as all close contacts with increased risk for invasive Hib disease (e.g. drug use, homelessness, chronic kidney/liver disease, malnutrition), close contacts in household-like settings, including housing facilities for PWUD or PEH and any close contacts who had regular contact with vulnerable people in the outbreak setting. Close contacts were eligible for post-exposure chemoprophylaxis up to 28 days after contact with a confirmed Hib case, as recommended for secondary case prevention in the UKHSA guidelines [9].

For close contacts presenting contraindications to rifampicin (e.g. comedication with specific pharmaceuticals such as polymerase inhibitors, non-nucleoside reverse transcriptase inhibitors, integrase inhibitors or protease inhibitors; pregnancy; severely impaired liver function; or undergoing methadone maintenance therapy) or with non-compliance concerns, ciprofloxacin was considered an appropriate alternative.

Post-exposure chemoprophylaxis could only be offered to four close contacts within the applied 28-day time frame from possible exposure to post-exposure chemoprophylaxis initiation. Three of these identified contacts were sharing apartments in the long-term housing facility for PEH where two confirmed cases lived. Contacts received a 4-day course of rifampicin (n = 3), or in cases with contraindications to rifampicin (n = 1) a single dose of ciprofloxacin (500 mg).

### Information campaign

Information materials about the outbreak in simplified German and with pictograms were prepared, explaining transmission routes and giving advice on personal protective measures. The SHAs and LHAs informed relevant facilities, such as housing facilities for PEH, addiction support centres with drug consumption facilities, clinics and specialised medical facilities within their area of responsibility, about the ongoing outbreak and short-term measures (post-exposure chemoprophylaxis) and medium to longer-term measures (vaccination). The developed information material was made publicly available and distributed among local actors in Hamburg (e.g. addiction support centres and supervised drug consumption facilities) [10].

### Vaccination

To protect vulnerable people and reduce ongoing Hib transmission, vaccination campaigns were initiated in Hamburg offering vaccinations to PWUD and staff of supervised drug consumption facilities and specialised medical practices. Since monovalent Hib vaccines can only be imported via international pharmacies and ordered for specific individuals by name in Germany, a pentavalent vaccine with a Hib component (DTPa-IPV-Hib) was used. People who were offered vaccination were informed about the off-label use and provided their consent. The size of the target group for vaccination could not be exactly estimated, with numbers of people living in Hamburg and reporting consumption of illegal psychoactive substances in the past year and/or experiencing homelessness in any form ranging between 5,000 and 40,000 [11,12]. People severely abusing crack / cocaine were considered to be at highest risk of contracting Hib, but no population estimates were available. Vaccinations started in early August 2025 in an established addiction support centre with supervised drug consumption in the city centre of Hamburg. By 31 August 2025, a total of 56 persons at increased risk of contracting Hib and 13 staff members had received a single dose of the pentavalent vaccine. An expansion of the vaccination campaign to specialised medical facilities, further supervised drug consumption facilities and facilities for PEH is ongoing.

Evidence on Hib vaccination was reviewed by STIKO, resulting in an update of the STIKO recommendations [13]. In addition to routine childhood vaccination and vaccination of immunocompromised persons with asplenia, Hib vaccination is now also recommended for people aged 5 years and older with a medically justifiable increased risk of invasive Hib disease, e.g. due to drug use or homelessness, in the context of an outbreak.

## Discussion

Outbreaks of invasive Hib disease are rare in the post-Hib vaccine era and, globally, have previously only been reported in children. This is the first reported outbreak of Hib among PWUD and PEH in Europe and resembles outbreaks in British Columbia, Canada, in 2022 and Alaska, Oregon and Washington, US, between 2023 and 2025 among PWUD and PEH [14,15].

Invasive Hib disease in adults typically affects individuals with splenic dysfunction, asplenia or other acquired or congenital conditions, impairing their immune defence against encapsulated bacteria [16]. However, this outbreak mainly affected male PWUD and PEH. Evidence from immunological studies suggests substance use and factors associated with precarious housing conditions can impair immunological competence [17,18]. Results from observational studies and animal models suggest that the use of crack / cocaine or heroin, the drugs most commonly reported by our cases, can considerably impair splenic function [19-21]. Inhalation of these drugs further weakens the mucosal barrier of the respiratory tract, while sharing drug equipment may contribute to enhanced transmission.

Further, unstable housing conditions are associated with malnutrition, chronic hepatitis C, excessive smoking and alcohol consumption. These factors further increase the risk of invasive bacterial infections [22-26]. Consistent with our observations, two outbreak investigations in British Columbia, Canada, in 2022 and Alaska, Oregon and Washington, US, between 2023 and 2025 also identified clusters of Hib in individuals without permanent housing, emphasising homelessness and associated comorbidities as suspected risk factors for invasive Hi infections [14,15].

A limitation of our investigation is that information on specific exposure was not routinely collected. This includes information on smoking status, mode of drug consumption, comorbidities and sharing of drug equipment.

Implementing public health responses can be challenging in this and similar outbreak settings. According to German and international recommendations [9,13,27], post-exposure chemoprophylaxis should be offered to close contacts as soon as possible after a Hib index case is confirmed, ideally within 7 days, but it can still be provided up to 14 days (Canadian recommendations) or 28 days (United Kingdom recommendations) after the last contact [9]. However, serotyping of Hi is not performed by primary diagnostic laboratories in Germany and Hi isolates must be sent to the NRZMHi for serotyping. Thus, Hib identification usually takes longer than 7 days. *Haemophilus influenzae* type b is likely also transmitted among asymptomatic carriers and re-exposure among PWUD and PEH in Hamburg is possible. Therefore, a more generous time span of 4 weeks for possible post-exposure chemoprophylaxis was applied following international recommendations [9]. This has subsequently been adopted by STIKO [13].

Following identification of close contacts, post-exposure chemoprophylaxis administration emerged as an additional challenge because of possible interaction of antibiotics, especially rifampicin [28], with consumed drugs and unclear adherence to a multi-day antibiotic regimen. Routinely recommended antibiotics for Hib- post-exposure chemoprophylaxis were therefore only partly suitable for identified contacts and expert consultations were held to identify appropriate alternatives.

Developing and agreeing on communication measures proved difficult. Despite the agreement of all stakeholders that the identified vulnerable population must be protected from further disease spread, uncertainty regarding the most appropriate communication strategy remained. Education and prevention measures in these groups require particular sensitivity. The authorities responsible for services for PEH in Hamburg were worried that overly direct approaches could deter affected individuals from using supervised drug consumption facilities or housing facilities supporting PEH. Further concerns were that this could result in PEH refraining from using other help and support services, in particular the winter aid programme, which was in place at the time of the outbreak detection. Therefore, the benefits and harms of different communication measures were carefully considered before the information campaign was finally initiated. However, despite all efforts for broad but sensitive communication, outbreak awareness is generally low in the affected population and cases continue to occur. Further, findings of genomic surveillance indicated ongoing transmission to other age groups and regions, as shown by the three genomically-related cases that had no epidemiological link to the outbreak. This highlights that measures implemented up to this point were insufficient to prevent further spread.

For the outbreak in Canada, the BCCDC promptly initiated outbreak control measures, including post-exposure chemoprophylaxis (administered to 92% of identified close contacts) and a vaccination campaign [14]. *Haemophilus influenzae* type b conjugate vaccines were administered to approximately 30% of the estimated vulnerable population (453/1,500 individuals). *Haemophilus influenzae* type b vaccination was concurrently offered with pneumococcal, influenza and COVID-19 vaccination and the outbreak could be contained. In contrast, Collins et al., reporting on a Hib outbreak in the US, described a prolonged outbreak and highlight difficulties in implementing an optimal vaccination strategy through limited resource availability, in particular in larger populations of PEH [15].

Vaccination was considered early in the outbreak in Germany. However, the absence of national vaccination recommendations for the affected population, limited market availability and licensing issues for adults delayed the implementation of a vaccination campaign. The outbreak triggered an update of the STIKO recommendations (published on 11 August 2025) on post-exposure chemoprophylaxis for close contacts of Hib cases and vaccinations in outbreak settings [13]. Vaccinations were implemented in the target population in early August 2025. However, difficulties in accessing eligible individuals (shortage of medical street workers), educating on off-label use and obtaining informed consent from the target population, bureaucratic difficulties with vaccine distribution and administration (distribution of vaccine doses to non-governmental organisations), legal concerns and uncertainties about the size of the at-risk population slowed their rollout.

As at 3 August 2026, the outbreak is ongoing, with the last Hib case reported in Hamburg on 26 June 2026 (genetically linked). Since 1 September 2025 and until the end of July 2026, one to four Hib cases associated with the described outbreak were reported per month, except for no cases in May and July 2026. By 3 August 2026, a total of 38 outbreak cases were identified. A molecular link was identified for 34 of these cases. In June 2026, NGOs, street workers and other stakeholders were involved in an information and vaccination campaign. Furthermore, in early July a four-day vaccination campaign was implemented in a public setting with low-threshold access for the affected population. By 4 August 2026 a total of 663 vaccinations have been administered to PWUD, PEH and facility staff following an intensified vaccination campaign since June 2026.

## Conclusions

In this report of an ongoing outbreak of Hib among PWUD and PEH in Hamburg, Germany, the consumption of inhaled drugs and the sharing of drug-use equipment were found as suspected risk factors for Hib. The event highlights that long-known bacterial pathogens can spread among susceptible populations that are not yet the traditional focus of surveillance or prevention programmes. Flexible and comprehensive surveillance systems are crucial for effective public health action. Genomic surveillance, including the submission of all bacteraemic Hi isolates to a reference laboratory for serotyping, is essential for detecting and investigating outbreaks in populations outside previously described risk groups. The outbreak prompted a timely update of STIKO recommendations, which now recommend Hib vaccination in outbreak settings among specific vulnerable populations. In addition to the existing indication groups (children under five years of age, people with an increased susceptibility to encapsulated bacteria (e.g. anatomical or functional asplenia)), this now also includes individuals aged five years or older who, in the context of an outbreak, face a medically substantiated increased risk of invasive Hib disease—for example, due to drug use, precarious housing situations or homelessness, chronic liver or kidney disease, or malnutrition. However, successful implementation of these recommendations, together with the integration of low-threshold structures (e.g. vaccination services) in collaboration with various stakeholders are necessary for containing the outbreak.

## Data Availability

Genomic data of the 45 Hib cases are available at https://pubmlst.org/organisms/haemophilus-influenzae with IDs 36358-36377 and 43059-43083.

## Supplementary Data

143000 Supplementary material
