# Supplementary material for: First year of ongoing invasive Haemophilus influenzae type b outbreak among middle-aged people who use drugs and people who experience homelessness in Hamburg, Germany, September 2024 to August 2025
Source: Euro Surveill. 2026 Sep 3;31(35):2600134. doi: 10.2807/1560-7917.ES.2026.31.35.2600134 (PMC13545523; doi:10.2807/1560-7917.ES.2026.31.35.2600134)
Supplement: Supplementary file 1 [file 26-00134_BUEHLER_Supplement.pdf]

This supplementary material is hosted by *Eurosurveillance* as supporting information alongside the article ‚First year of ongoing invasive *Haemophilus influenzae* type b outbreak among middle-aged people who use drugs and people who experience homelessness in Hamburg, Germany, September 2024 to August 2025‘, on behalf of the authors, who remain responsible for the accuracy and appropriateness of the content. The same standards for ethics, copyright, attributions and permissions as for the article apply. Supplements are not edited by *Eurosurveillance* and the journal is not responsible for the maintenance of any links or email addresses provided therein.

Supplement S1. Overview of consulted recommendations regarding chemoprophylaxis in an outbreak setting of Germany (STIKO), Canada (BCCDC) and the United Kingdom (UKHSA)

	**Germany (1)** (valid until 20 August 2025)	**Canada (2)**	**United Kingdom (3)**
**Background/ specifications**	Since transmission is rare in cases of infection with Hi serotypes other than Hib, post-exposure prophylaxis is not recommended in these cases.<br>Prophylaxis should be started as early as possible, but no later than 7 days after the onset of disease in the index case.	Chemoprophylaxis is only recommended for cases/contacts of Hib, not for other serotypes<br><br><u>Definition of a contact of a case of invasive Hib disease:</u><br>A person residing with the case of invasive Hib disease OR a person who has spent 4 or more hours per day with the case for at least 5 of the 7 days preceding the day of hospital admission of the case. It is assumed that when children have spent 4 or more hours together per day, they are likely to have napped and/or eaten together, which increases transmission risk<br><br>When indicated, chemoprophylaxis should be initiated as soon as possible without first establishing whether the individual has nasopharyngeal carriage of the organism. If more than 14 days have passed since the last contact with the index case, the benefit of chemoprophylaxis is likely to be decreased.	Definition of household contact: the definition of a household contact is “any individual who has had prolonged close contact with the index case in a household type setting during the 7 days before the onset of illness”. Examples of a household contact include living or sleeping in the same house, boyfriends or girlfriends, and sharing a dormitory, flat or hospital ward* with the index case. Other types of contact (for example, at work or school) would not be considered close contact, but each situation should be considered on its own merit, particularly if a vulnerable contact is involved and a close contact group can be clearly defined.<br><br>* Comment from the guideline authors: wards may have different constructions/architecture depending on the hospital/country – and this recommendation may not apply to all settings

**To whom**	Chemoprophylaxis is recommended for all household members of patients with invasive Hib infection who are at least 1 month old and have close (face-to-face) contact, if an unvaccinated or inadequately vaccinated child under 5 years of age or a person with relevant immunodeficiency or suppression lives in their household.<br>In addition, unvaccinated children under 5 years of age should also receive appropriate chemoprophylaxis if they have had close contact (face-to-face) with a person suffering from invasive Hib disease in a community setting.<br><br>If ≥ 2 Hib cases have occurred in a community setting for young children within approximately 2 months and the setting cares for unvaccinated or insufficiently vaccinated children, chemoprophylaxis is recommended for all children, regardless of vaccination status and age, as well as for caregivers of the same group.	Chemoprophylaxis is recommended for<br>(1) All household contacts, regardless of age, in the following circumstances:<br>a. at least 1 contact younger than 4 years of age who is unimmunized or incompletely immunized for age<br>b. a child younger than 12 months of age if the child has not received the primary series of three doses<br>c. an immunocompromised child regardless of that child's Hib immunization status (i.e., even if fully immunized).<br>(2) Preschool/day care contacts (including staff), regardless of age, when 2 or more cases of invasive Hib disease have occurred within 60 days among attendees and unimmunized or incompletely immunized children are attending.<br>(3) The case, if younger than 2 years of age or is a member of a household with a susceptible contact, and who had been treated with a regimen other than cefotaxime sodium or ceftriaxone sodium. Chemoprophylaxis usually is provided just before discharge from hospital<br><br>Chemoprophylaxis MAY be considered in the following situations at the discretion of the Medical Health Officer:<br>(1) All household contacts of the case when at least one contact is a child of any age with immunodeficiency, sickle cell disease, asplenia, or leukemia<br>(2) Health care workers who have administered mouth-to-mouth resuscitation to the case<br>A small well-defined population deemed at high risk of severe disease following the occurrence	The index cases and all household contacts of the index case with confirmed invasive Hib disease should receive chemoprophylaxis.<br>Chemoprophylaxis should be offered to the index case and all eligible contacts up to 4 weeks after onset of illness in the index case as soon as Hib is confirmed in the index case.<br>For settings where a group of children with high levels of contact approaching those in the household can be defined – for example, a small number of children attending the same child-minder for several hours each day – offering antibiotic prophylaxis to the close contact group should be considered, including staff.<br>In case of an outbreak (2 or more cases of Hib disease within 120 days), as well as the above, chemoprophylaxis should also be offered to all room contacts, including staff

		of one or more cases in the context of likely clonal transmission	
**Drug and regimen**	Rifampicin orally for 4 days is the drug of choice for prophylaxis (age-dependent dosage). Since the administration of rifampicin is contraindicated in pregnant women, they may receive ceftriaxone (1 x 250 mg IM).	Rifampicin given orally for 4 days is the prophylaxis of choice:<br>• Infants < 1 month of age: 10 mg/kg per dose PO once daily x 4 days<br>• Children ≥ 1 month of age: 20 mg/kg (to maximum 600 mg) per dose PO once daily x 4 days<br>• Adults (≥ 18 years of age): 600 mg PO once daily x 4 days<br><br>Ceftriaxone (IM) may be used as an alternative prophylaxis for pregnant people, individuals for whom rifampicin is contraindicated or who cannot tolerate oral medication.<br>• Adults and children ≥ 12 years of age: A single dose of 250 mg, or up to 1 gram once a day for two days, given IM<br>Children < 12 yrs: A single dose of 125 mg, or up to 50 mg/kg once a day for two days, given IM	Rifampicin at a dose of 20 milligrams per kilogram (mg/kg) (maximum 600mg) once a day for 4 days for adults and children older than 3 months is the prophylaxis of choice for eliminating carriage in the index case and among household contacts<br>Infants younger than 3 months should receive 10 mg/kg once a day for 4 days<br>Pregnant and breastfeeding women should also receive rifampicin prophylaxis if there is a vulnerable individual among the household contacts because the benefits of providing chemoprophylaxis to all household contacts, including pregnant and breastfeeding women, outweigh any potential risks.<br>Once daily intravenous or intramuscular ceftriaxone (50 mg/kg in children younger than 12 years, one gram for older children and adults) once a day for 2 days is, therefore, recommended as an alternative agent in an individual who is unable to tolerate or develops an adverse reaction to rifampicin.<br>Treatment courses of oral ciprofloxacin (500mg in adults and children older than 12 years, 250mg for children aged 5 to 12 years, 125mg for children 2 to 4 years) twice a day for 5 days or azithromycin (10 mg/kg, maximum dose 500mg) once a day for 3 days may be used as alternatives, but their effectiveness in eradicating Hib colonisation among healthy individuals has not been determined. The use of ciprofloxacin in paediatrics was previously limited because of concerns regarding irreversible quinolone-induced arthropathy documented in juvenile animal models, although such effects have not been observed despite extensive use. Ciprofloxacin suspension is

			licensed for other indications in children over one year of age and has been recommended for prophylaxis against meningococcal disease in adults and children.

Supplement S2. Overview of consulted recommendations regarding vaccination in an outbreak setting of Germany (STIKO), Canada (BCCDC) and the United Kingdom (UKHSA)

	**Germany (1)** (valid until 20 August 2025)	**Canada (2)**	**United Kingdom (3)**
**Background**			The 2024 guidance now recommends public health action for all confirmed cases of Hib. This guidance supersedes previous recommendations for public health action only if there is a child under 10 years of age or a vulnerable individual in the household.
**To whom**	Completing Hib immunisation series in children < 5 years is recommended	Hib disease has not been recognized as a cause of community-based outbreaks until recently in BC, and this phenomenon is thought to be associated with a Communicable Disease Control Haemophilus Influenzae type b clonal strain of the organism affecting adults, many of whom are homeless/ under-housed.<br>Following the occurrence of an invasive case in an adult associated with the homeless/underhoused population, social network contacts including those in shared accommodation such as a shelter or single room occupancy housing and accessing common services for these populations may be offered Hib vaccine at the discretion of the regional MHO; workers at these locations may also be offered Hib vaccine. Offer Hib vaccine regardless of prior Hib vaccine receipt. For those with a record of Hib vaccine within the past 12 months, an additional dose is not needed	Unimmunised and partially immunised children younger than 10 years in the household should complete their immunisations according to the UKHSA guidance.

References:

1. Ständige Impfkommission. Empfehlungen der Ständigen Impfkommission (STIKO) beim Robert Koch-Institut. Epid Bull. 2025(4):1–75.
2. British Columbia Centre for Disease Control. Communicable Disease Control Manual, Haemophilus influenzae type b. Vancouver; 2024.
3. UK Health Security Agency. Revised recommendations for the prevention of secondary Haemophilus influenzae type b (Hib) disease. London; 2023.
